# Fracture Healing in Two Adult Patients With Hypophosphatasia After Asfotase Alfa Therapy

**DOI:** 10.1002/jbm4.10052

**Published:** 2018-05-17

**Authors:** Philostratos Klidaras, Jacqueline Severt, Deborah Aggers, Jason Payne, Paul D Miller, Steven W Ing

**Affiliations:** ^1^ Skaggs School of Pharmacy and Pharmaceutical Sciences Aurora CO USA; ^2^ Department of Medicine Case Western Reserve Cleveland OH USA; ^3^ Foothills Urology, PC Golden CO USA; ^4^ Department of Radiology The Ohio State University Wexner Medical Center Columbus OH USA; ^5^ Colorado Center for Bone Research at Panorama Orthopedics and Spine Center University of Colorado Health Sciences Center Golden CO USA; ^6^ Division of Endocrinology, Diabetes, and Metabolism The Ohio State University Wexner Medical Center Columbus OH USA

**Keywords:** HYPOPHOSPHATASIA, ASFOTASE ALFA, FRACTURE

## Abstract

Infants and children with hypophosphatasia (HPP) treated with asfotase alfa show improvement in bone mineralization and motor function, but it is unclear whether the medication can affect fracture healing in adult HPP patients. We present the course of fracture healing in two adults with HPP on enzyme replacement. Case 1 is a 41‐year‐old female with infantile‐onset HPP who was wheelchair‐bound due to a nonhealing tibial fragility fracture sustained 3 years before and also had nonhealing femoral pseudofracture sustained 17 years before starting asfotase alfa therapy in December 2015. One month after medication initiation, she underwent elective osteotomy of tibia and fibula with intramedullary nail fixation. After 3 months of enzyme replacement, she was full weight‐bearing and radiographs demonstrated callus formation at osteotomy sites, and at 11 months of therapy, radiographs showed union of the osteotomies. By 11 months of asfotase alfa therapy, there was near resolution of the femoral pseudofracture without interval surgery at this site. Case 2 is a 61‐year‐old male who showed nonunion of a fragility fracture of the right femur 8 years prior, intramedullary nail fixation 6 years prior, and stress fracture of the left femoral diaphysis sustained 1 year before starting asfotase alfa in October 2015. A trial of teriparatide was unsuccessful in healing of these fractures. On asfotase alfa, radiographs revealed interval healing of the left femur fracture after 12 months and complete healing of the right femur fracture and near resolution of the left femur fracture after 16 months of medical therapy. These two adult patients with HPP showed significant clinical and radiographic improvements in a total of four recalcitrant fractures on enzyme replacement therapy with asfotase alfa. © 2018 The Authors. *JBMR Plus* published by Wiley Periodicals, Inc. on behalf of American Society for Bone and Mineral Research.

## Introduction

Hypophosphatasia (HPP), also known as Rathbun disease, is a rare, heritable inborn error of metabolism that has both autosomal dominant and recessive inheritance patterns due to loss‐of‐function mutations in the alkaline phosphatase gene (*ALPL*) located on the short arm of chromosome 1, which encodes the tissue nonspecific isozyme of alkaline phosphatase (TNSALP).^1^, ^2^, ^3^, ^4^ Clinical features of HPP include low serum alkaline phosphatase (ALP) activity, defective mineralization of bone, and elevated levels of ALP substrates such as pyridoxal‐5‐phosphate (vitamin B6). Skeletal manifestations include bone pain, pathologic fractures, poor fracture healing, and poor dentition.

In October 2015, the FDA approved asfotase alfa, a bone‐targeted human recombinant TNSALP replacement therapy for patients with perinatal‐, infantile‐ and juvenile‐onset HPP.^3^ This recombinant glycoprotein contains the catalytic domain of TNSALP, human immunoglobulin G_1_ Fc domain, and a deca‐aspartate peptide, which attaches to bone. Clinical studies of asfotase alfa have focused on a pediatric‐age population with fewer data in adults.^5^, ^6^ Asfotase alfa‐treated infants and children with HPP show improvements in bone mineralization and respiratory and motor function.^7^ Despite evidence for such improvement, it remains unclear whether the medication affects bone mineralization or fracture healing in adults with HPP. We present two cases of adults with HPP who initiated asfotase alfa therapy, suggesting that may aid in fracture healing.

## Case 1

**Figure 1:**
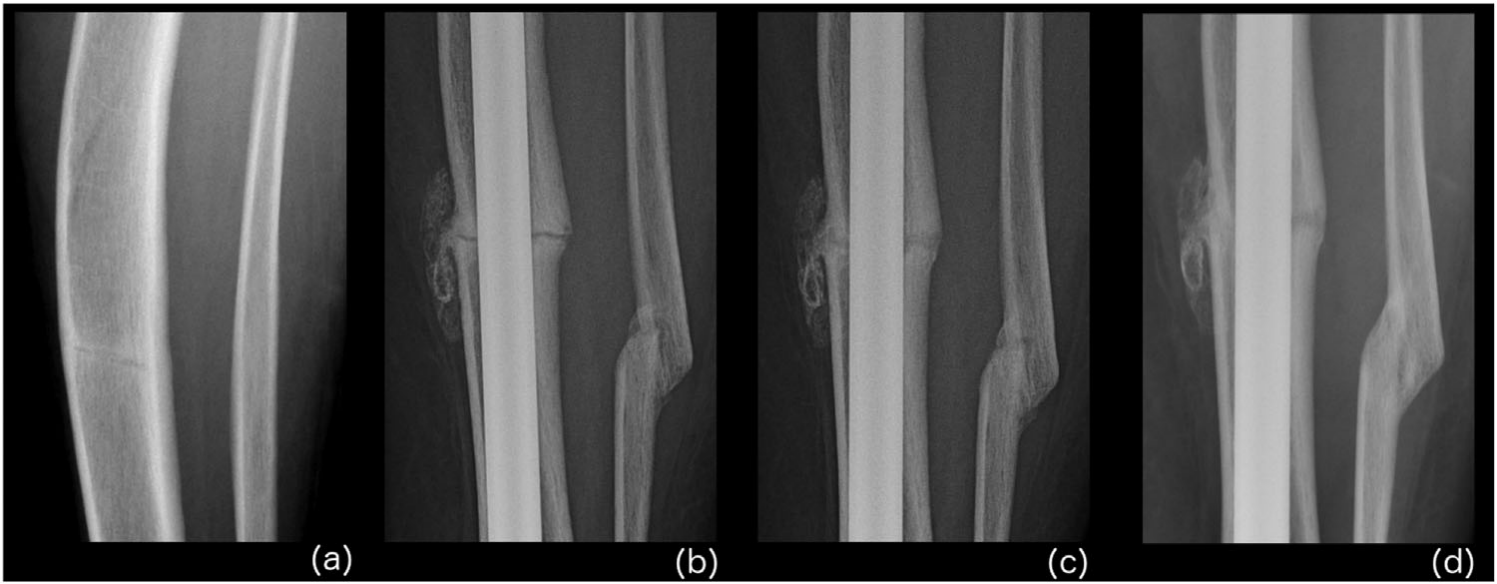
Case 1: Radiograph series of left tibia‐fibula after osteotomy and intramedullary nail (IMN) fixation. (*A*) Three years after left tibial fracture; before replacement therapy and operative treatment. (*B*) One month after elective osteotomy; 2 months after asfotase alfa initiation. Some callus formation at the osteotomy site. (*C*) Five months post‐op; 6 months on medication. Definite increase in callus formation. (*D*) Ten months post‐op; 11 months on medication. Tibial and fibular osteotomies appear to have gone on to union.

A 41‐year‐old female was diagnosed with infantile‐onset HPP at the age of 5 months, presenting with recurrent pneumonia and rib fractures. She had additional fractures of the extremities, rickets, and short stature. In 1998, the patient was found to have bilateral subtrochanteric femoral shaft pseudofractures after a fall down a flight of stairs and was treated with intramedullary nail (IMN) fixation at the time. In 2012, she suffered a left tibial fragility fracture from stepping off a sidewalk curb. She was initially treated with a cast, walking boot, cane, walker, and eventually was full non‐weight‐bearing due to fracture nonhealing (Fig. 1 *A*). Her height, 138.4 cm (54.5″), was less than mid‐parental height of 174 cm (68.5″). She was edentulous. Baseline labs in November 2013 included ALP 8 U/L (38–126) and vitamin B6 2450 mcg/L (5–50). Gene testing of *ALPL* showed compound heterozygous mutations c.526G>A (p.Ala176Thr) and c.1132G>C (pAsp378His).

In December 2015, she started asfotase alfa, 1 mg/kg subcutaneous injection six times per week. One month later, she underwent elective osteotomy of the left tibia and fibula with IMN fixation. Serial radiographs after the procedure demonstrated callus formation by 1 month postoperatively (2 months after initiating enzyme replacement therapy; Fig. 1 *B*). The patient began physical therapy shortly after surgery and was full weight‐bearing by 2 months post‐op. By 5 months post‐op (6 months after initiating astatase alfa), there was a definite increase in bridging callus formation (Fig. 1 *C*), and the patient was able to ambulate independently at this time. By 11 months post‐op, radiographs demonstrated union of the left tibia and fibular osteotomies (Fig. 1 *D*). Notably, at this time she reported greatly improved walking tolerance, up to distances of 4 miles. At physical therapy sessions, her subjective bone pain scores were documented to improve from 8 to 10 (on a 1 to 10 scale) at the start of asfotase alfa to 3 to 6 by 9 months of replacement therapy.

**Figure 2:**
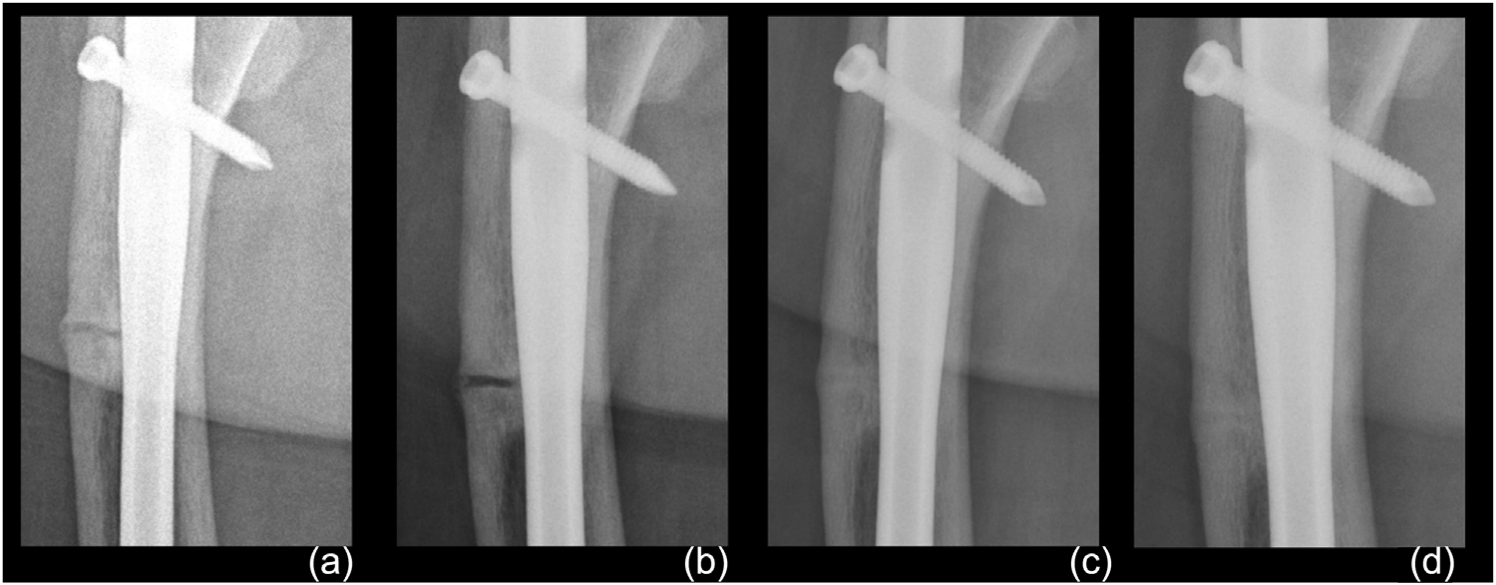
Case 1: Radiograph series of right subtrochanteric femoral pseudofracture. (*A*) Right femur pseudofracture 12 years after identification; before asfotase alfa treatment. (*B*) Stable 17 years after fracture; 1 month before asfotase alfa therapy. (*C*) Eleven months of uninterrupted asfotase alfa treatment; radiograph demonstrates progressive healing of femoral pseudofracture. (*D*) Fourteen months on medication; continued bone healing.

Moreover, the subtrochanteric femoral pseudofractures showed little remodeling at the fracture line over the 17 years before starting asfotase alfa (Fig. 2 *A*, *B*). By 11 months of replacement therapy, radiographs showed marked healing with near resolution of the fracture line (Fig. 2 *C*) and continued bone healing at 14 months of replacement therapy (Fig. 2 *D*).

## Case 2

A 61‐year‐old male presented with a fragility fracture of the right femur in May 2006, which required intramedullary rod placement in December 2008. He had persistent pain and a waddling gait. Serial radiographs demonstrated nonunion of the fracture 8 years later despite operative intervention. In 2014, he complained of left knee pain, as well as difficulties with gait and balance and saw another orthopedic surgeon. Radiographs demonstrated a new fracture of the left femoral diaphysis as well as the continued nonunion of the previous right femur fracture. He declined operative intervention because he was dissatisfied with the lack of healing of the contralateral fracture and sought another opinion.

Physical examination showed poor dentition and a waddling gait. A complete laboratory workup was completed and significant for ALP 6 U/L (39–177), phosphorus 5.5 mg/dL (2.5–4.5), and vitamin B6 >100 µg/L (5.3–46.7). Based on these results, HPP was suspected and genetic testing confirmed HPP with compound mutations c.874C>A (p.Pro292Thr) and c.1195G>A (p.Ala399Thr).

In March 2015, the patient initiated a course of teriparatide to assist with fracture healing. After 6 months, he discontinued this treatment, as he was unable to tolerate the side effects of the drug and X‐rays did not demonstrate interval healing.

**Figure 3:**
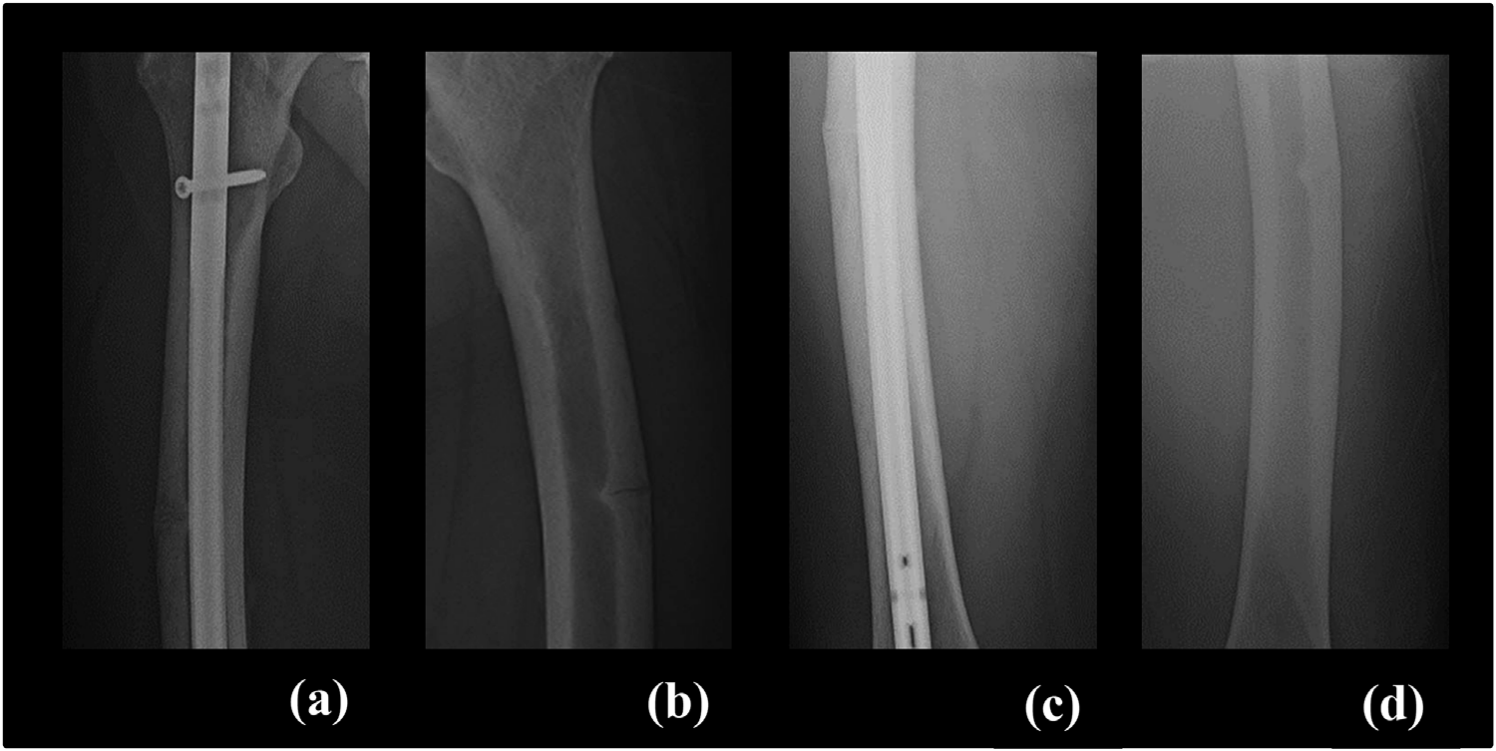
Case 2: Radiograph series of left and right femoral fractures. (*A*) Right femur 9 years after fracture; before asfotase alfa treatment. (*B*) Left femur; before asfotase alfa treatment. (*C*) Right and (*D*) left femur 16 months on medication.

The subject received first dose of asfotase alfa in October 2015. In November 2016, radiographs revealed interval healing of the left femur fracture. In February 2017, bilateral radiographs demonstrated complete healing of the right femur and near resolution of the left femur (Fig. 3), and the patient reported significantly less pain with ambulation, improvement in gait, and overall improved quality of life.

## Discussion

We report two adult patients with HPP who started asfotase alfa therapy with resulting healing of a total of four fractures. The patients demonstrated remarkable bone healing on replacement therapy when compared with the paucity of healing after >3, 8, and 17 years of previously sustained tibial and femur fractures. On enzyme replacement, radiographs showed tibial bone healing in Case 1 at a rate just slightly slower than the average range observed in normal patients, who are expected to show some callus formation after tibial osteotomy by 4 to 6 weeks and complete fracture healing by 5 to 8 months. Some callus formation appeared at the osteotomy site by 1 month post‐op, 2 months into asfotase alfa replacement therapy. Callus formation was found by 5 months post‐op, and osteotomy site has progressed to union by 10 months post‐op. These findings are remarkable considering the patient's lack of progression in bone healing over 3 years since tibial fracture and before enzyme replacement therapy. It remains possible that the second tibial surgery was responsible for bone healing and therefore might confound the interpretation of benefit due to medication effect. However, a nonhealing femoral pseudofracture persisted for 17 years after femoral IMN fixation and remarkably progressed to union in Case 1 and bilateral femoral fractures in Case 2 showed healing once on enzyme replacement. Notably, none of these three fractures were treated with further surgery. With three instances of fracture healing without further surgery, we argue the healing of tibial fracture in Case 1 is most likely attributable to the medication effect as well. Moreover, on asfotase alfa, there were significant improvements in physical function, pain, and quality of life.

Measurement of physical function, pain, and quality of life and time course of improvement were not tracked with specific metrics, a limitation of this work. Recently, guidance has been provided, including 6‐minute walk test, dynometry, observational gait analysis, and Wong‐Baker FACES Pain Rating Scale, EQ‐5D‐5L, and SF‐36 for adult HPP patients treated with asfotase alfa.^8^

Although healing of the fibular osteotomy (Fig. 1) may be considered as malunion, progressive bridging callus at both sites is more important to be healed correctly and the alignment of the healing osteotomy is largely irrelevant. Moreover, given the small contribution to weight‐bearing and location in the shaft of the fibula, this malunion is acceptable. Most fibula fractures in this location can be treated conservatively provided there is no ankle injury and do well.

These patients have continued more than 14 to 16 months of uninterrupted asfotase alfa therapy, experiencing continued bone healing without complications from the treatment to date. In summary, these two cases highlight significant clinical and radiographic improvement in bone healing after long bone fractures in adult patients with HPP on asfotase alfa therapy.

## Disclosures

SWI and PDM have grant funding from Alexion Pharmaceuticals. All other authors state that they have no conflicts of interest.
